# The molecular basis of memory. Part 3: tagging with “emotive” neurotransmitters

**DOI:** 10.3389/fnagi.2014.00058

**Published:** 2014-04-07

**Authors:** Gerard Marx, Chaim Gilon

**Affiliations:** ^1^MX Biotech Ltd.Jerusalem, Israel; ^2^Department of Organic Chemistry, Institute of Chemistry, Hebrew UniversityJerusalem, Israel

**Keywords:** metal complex, *cuinfo*, neurotransmitters, emotion, mentation

## Abstract

Many neurons of all animals that exhibit memory (snails, worms, flies, vertebrae) present arborized shapes with many varicosities and boutons. These neurons, release neurotransmitters and contain ionotropic receptors that produce and sense electrical signals (ephaptic transmission). The extended shapes maximize neural contact with the surrounding neutrix [defined as: neural extracellular matrix (nECM) + diffusible (neurometals and neurotransmitters)] as well as with other neurons. We propose a *tripartite* mechanism of animal memory based on the dynamic interactions of splayed neurons with the “neutrix.” Their interactions form cognitive units of information (*cuinfo*), metal-centered complexes within the nECM around the neuron. Emotive content is provided by NTs, which embody molecular links between physiologic (body) responses and psychic feelings. We propose that neurotransmitters form mixed complexes with *cuinfo* used for tagging emotive memory. Thus, NTs provide encoding option not available to a Turing, binary-based, device. The neurons employ combinatorially diverse options, with >10 NMs and >90 NTs for encoding (“flavoring”) *cuinfo* with emotive tags. The neural network efficiently encodes, decodes and consolidates related (entangled) sets of *cuinfo* into a coherent pattern, the basis for emotionally imbued memory, critical for determining a behavioral choice aimed at survival. The *tripartite* mechanism with tagging of NTs permits of a causal connection between physiology and psychology.

## Introduction

**Figure 1:**
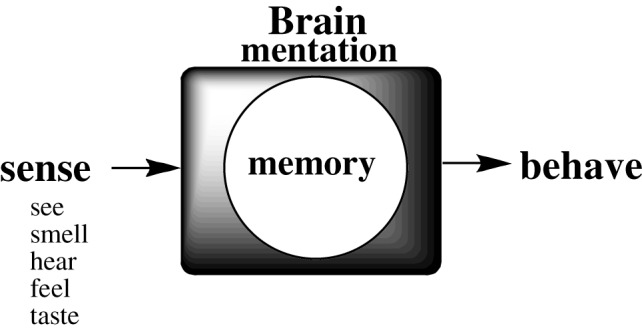
**Schematic of the process whereby an external stimulus is remembered to determine whole body response, critical for survival**.

The neural circuitry of the brain has been likened to a biological computing device. But the process whereby a physiologic process (stimulus sensation) transforms into a psychical sensation (such as emotionally-tinged memory), which determines physical response to immediate stimuli, remains mysterious (Figure 1).

Each of the senses receives environmental stimuli (input) which are transformed into a synaptic cognitive information (cog-info) signals, which are somehow encoded and stored somewhere in the brain, later to be decoded (recalled), to determine a behavioral choice based on recalled experience. Much has been speculated in philosophical term (Romanes, 1883; James, 1884; Langer, 1967; Meshulam et al., 2011) and on the basis of biologic observations (Squire and Kandel, 2008; Kandel, 2009; Garcia-Lopez1 et al., 2010; DeFelipe, 2011; Murtya et al., 2011; Emmons, 2012; Jarrell et al., 2012; Hirano et al., 2013; Strausfeld and Hirth, 2013; Wright et al., 2013), but molecular details for the mentation of memory by neural animals are lacking.

Q: Does the brain operate like a Turing machine (Boole, 1853; Turing, 1950)?

**Figure 2:**
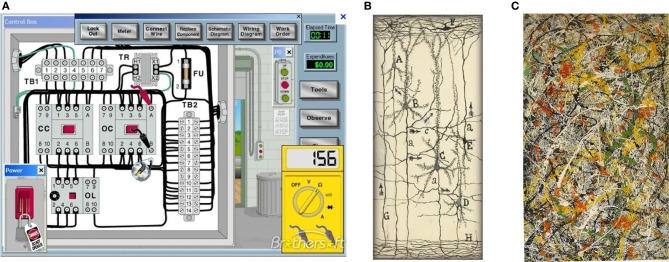
**(A)** Circuit for electric motor. (Note the insulating spaces between wires and components). **(B)** Cajal drawing of a neural circuit (from Garcia-Lopez1 et al., 2010; DeFelipe, 2011). (Note the empty spaces around neurons) (with permission). **(C)** Painting by Jackson Pollok (Boole, 1853) that more closely represents neurons enmeshed within the nECM. (No empty spaces). (with permission).

A: Computer and machine circuits (Figure 2A) operate in dry condition. Wires in an electric circuit are insulated from one another by plastic, non-conducting, coatings and air gaps (or vacuum), to prevent short circuits.

By contrast, we previously pointed out that neurons are enclothed in a wet, electrically conducting hydrogel (nECM) with many component glycosaminoglycans (GAGs) and proteins (Marx and Gilon, 2012, 2013). The intimate contacts of the extended neural surface with the nECM permits iontophoretic/piezo-electric/visco-elastic actuators on the neural surface to metamorph cog-info into *cuinfo*. Images of “naked” neurons suspended in vacuous space are misleading, in that they ignore the nECM and the dopants (NTs and neurometals) distributed therein. Cajal did not perceive their roles in neural mentation (Figure 2B; see Garcia-Lopez1 et al., 2010). Rather, the intuitive painting of Pollock (1997) more closely represents the physical circumstances of neurons enclothed within a biogel lattice (Figure 2C).

## Encoding emotions

Neural memory recalls *emotive* as well as *objective* qualities. As conceived by the philosopher William James and other philosophers (Romanes, 1883; James, 1884; Langer, 1967; Meshulam et al., 2011), emotions have physical correlates.

Q: What kind of molecular structure or process endows memory with emotive quality?

A: Possibly, neurotransmitters (NTs) are involved. They eliciting a range of physiologic and psychic responses and most of them bind to metals.

We point out that the NTs are a class of molecules synthesized and secreted by neurons that elicit emotive reactions, concomitant with physiologic responses. Thus, NTs can be considered as the molecular embodiments of emotions. In that they have strong affinity to metals, they can form ternary complexes with metals, as exemplified by the binding of bilirubin to albumin (Marx, 1984). We propose that the NTs (Hughes and Zubek, 1956; Colburn and Maas, 1965; Boggess and Martin, 1975; Chandra et al., 1980; Ludlam et al., 1980; Sigel and Martin, 1982; Jolles, 1983; Coffman and Dunn, 1988; Flood et al., 1990; Jefferys, 1995; Velez-Pardo et al., 1995; White and Rumbold, 1988; Buhot et al., 2000; Reith, 2002; Shaik, 2003; Álvarez and Ruarte, 2004; Siegel et al., 2005; Kroval et al., 2006; Marazziti et al., 2006; Neumann, 2007; Wyttenbach et al., 2008; Paoletti et al., 2009; van der Burgt et al., 2009; Burbach, 2010; Dere et al., 2010; Guastella et al., 2010; Brady et al., 2011; Lesburguères et al., 2011; Beets et al., 2012; García et al., 2012; Garrison et al., 2012; Ma et al., 2013; Pitt et al., 2013; Yanagita et al., 2013) provide the neural net with a new mode of processing (mentating) cognitive information (cog-info) not available to a binary Turing machine.

## *tripartite* mechanism

To rationalize the phenomenon of biologic memory in physical-chemical terms, we have proposed (Marx and Gilon, 2012, 2013) a *tripartite* mechanism comprising 3 physiologic compartments:

- Neuron—elongated cell in synaptic and non-synaptic contact with others
- nECM—an anionic biogel lattice surrounding the neuron
- dopants—neurometals and neurotransmitters (NTs)—metals (e.g., Al^+3^, Ca^+2^, Co^+2^, Cu^+2^, Fe^+2/3^, Mg^+2^, Mn^+2/3^, Zn^+2^) and small molecular modulators, distributed in the nECM.

Though the term “space” is often used to refer to the neurons' environment, it is not quite correct. The neurons are not naked, floating in space. Rather, they are suspended (enmeshed) in a matrix composed of glycosamino-glycans (GAGs) and proteins (such as tenascins, and laminin), referred to as “nECM.” Their shape (highly elongated with many dendrites, splayed, arborized) exposes the large surface to intimate contact with the nECM, through which chemical, as well as electrical, signaling occur.

Just like all other physiologic processes, mentation must be biochemically based. All three of the above compartments are involved in transforming (encoding) cognitive information (cog-info) incoming from the senses, into [nECM:metal] complexes, the molecular correlates of *cognitive units of information* (*cuinfo*), like computer bits. The incoming cog-info is transferred to the brain via into synaptic and non-synaptic networks. But what happens there?

We generalize a biochemical processes and notations, which feature sets of metal-centered complexes (*cuinfo*) which can undergo different types of redox, tagging and cross-linking reactions, thereby modulating the dielectric properties, viscoelasticity and stability of local, molecular ensembles. The neuron is atuned to such nECM ensembles and can thereby chemically affect/sense (encode/decode) cog-info.

Focusing on the neurotransmitters (NTs) shown in Table 1, all present a variety of metal complexing moieties called ligands (e.g., glycine, glutamate catechol amines, neuropeptides, adenosine) that can form mixed complexes with *cuinfo.* Activated neurons release vesicles containing NTs along with neurometals (M^+*v*^; such as Ca^+2^, Cu^+2^, Fe^+3^, Mn^+2^, Zn^+2^, etc.) into the nECM of the synaptic cleft and other extracellular locations, permitting the formation of ternary complexes [nECM: M^+*v*^:NT]. Some larger one permit coordination with more than 1 metal centered *cuinfo* (bidendate, tridendate).

**Table 1:**
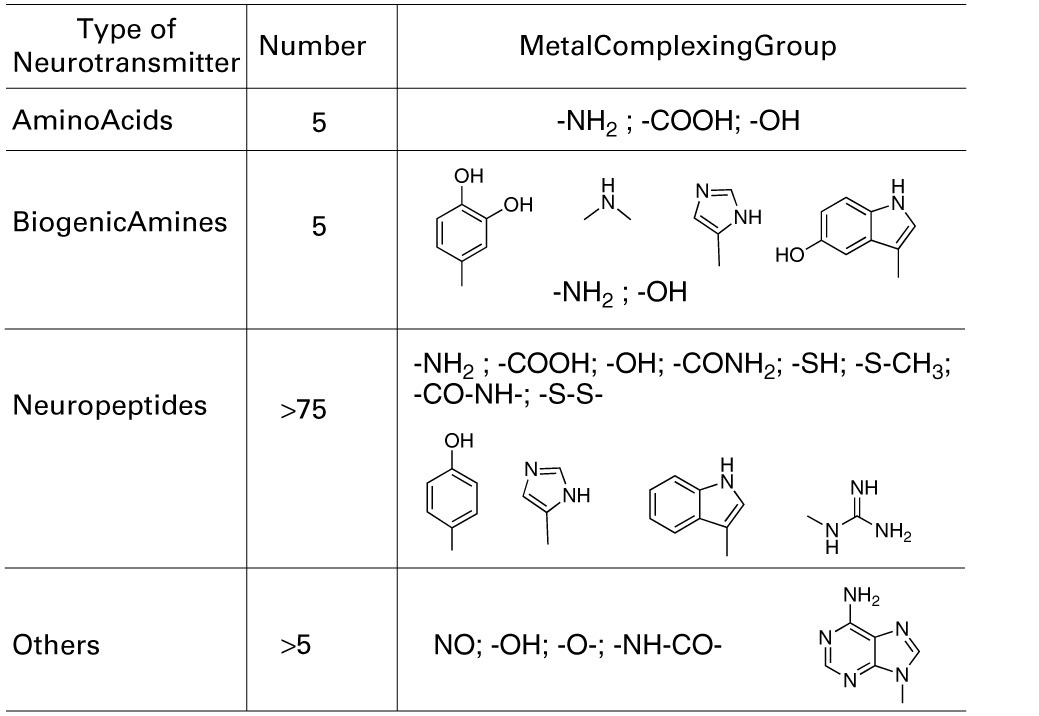
**Metal complexing neurotransmitters**.

Types and structures (Hughes and Zubek, 1956; Colburn and Maas, 1965; Boggess and Martin, 1975; Chandra et al., 1980; Ludlam et al., 1980; Sigel and Martin, 1982; Jolles, 1983; Coffman and Dunn, 1988; Flood et al., 1990; Jefferys, 1995; Velez-Pardo et al., 1995; White and Rumbold, 1988; Buhot et al., 2000; Reith, 2002; Shaik, 2003; Álvarez and Ruarte, 2004; Siegel et al., 2005; Kroval et al., 2006; Marazziti et al., 2006; Neumann, 2007; Wyttenbach et al., 2008; Paoletti et al., 2009; van der Burgt et al., 2009; Burbach, 2010; Dere et al., 2010; Guastella et al., 2010; Brady et al., 2011; Lesburguères et al., 2011; Beets et al., 2012; García et al., 2012; Garrison et al., 2012; Ma et al., 2013; Pitt et al., 2013; Yanagita et al., 2013).

With the exception of acetylcholine and muscarine, which are true cationic entities due to their tetrasubstituted ammonium moiety, most NTs are electron donors, behaving as effective metal complexants (ligands) (Table 1). An individual NT can be considered to embody an “emotive” signal, if it elicits physiologic responses (pulse, breathing, dilation of blood vessels and pupil, erection, sweating, etc), as well as corresponding psychic reactions (attention, anxiety, anger, fear, hunger, pain, love, etc.), which are remembered. Chemically, an electron-rich NT molecule diffusing in the nECM can bind to a metal-centered *cuinfo*, confers an emotive tag to the ternary (mixed) complex, resulting in a tagged, *cuinfo:NT*. The stability of such complexes depends on the valency of the metal cation and binding affinity of the components (pK_D_). Monovalent metal (Na^+^, K^+^, Li^+^, Cs^+^) complexes are relatively unstable; the resultant *cuinfo* tend to rapidly disintegrate. Small mono-dendate NTs bind to a single *cuinfo*; larger ones are polydendate and could bind to multiple *cuinfo*, thereby literally “entangling” them. Table 2 below organizes the metal-complexing NT which have been shown to induce physiologically-linked psychic responses to stimuli (Table 2), are also imprinted in memory.

**Table 2:** **Bio-modulators (also called NTs) of physiologic responses to stimuli, which simultaneously elicit both physiologic responses and psychic (emotions) feelings, which also encode the stimuli, aiding the recall (memory)**.

**Modulators (neurotransmitters)**	**Metal complexing ligands**	**Physiologic reactions**	**Psychic effects emotions**
Acetylcholine (AcChol)	NO	Breathing	Anxiety
Epinephrine (EPI)	YES	Blinking	Aggression
Serotonin (SER)	YES	Blood pressure	Awareness
Histamine (HIS)	YES	Coughing	Depression
Nicotine	YES	Crying	Fear
Muscarine	NO	Dilation of pupil	Hate
Amino acids	YES	Drooling	Heat
>75 neuropeptides	YES	Erection	Hunger
		Evacuation	Joy
		Fever	Love
		Goose-bumps	Pain
		Heart beat	Sadness
		Itching	Sexdrive
		Orgasm	
		Pulse	
		Salivation	
		Spasms	
		Sweating	
		Tremors	
		Urination	
		Vasodilation	
		Vomiting	

## Iconography

**Figure 3:**
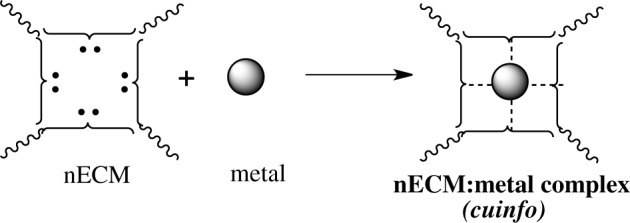
**Iconographic representation of formation of anECM:metal complex (*cuinfo*).** The chelating node (address) within the nECM is presented as square electron-rich hole fixed within the nECM lattice, with 2 dots representing ligands available for capturing a metal. The metal-bonded to the complexing group electrons is indicated by a dotted line, within the *cuinfo*. It can serve as a binding focus for metabolites and neurotransmitters. The nECM array with metal complexes is called neutrix.

**Figure 4:**
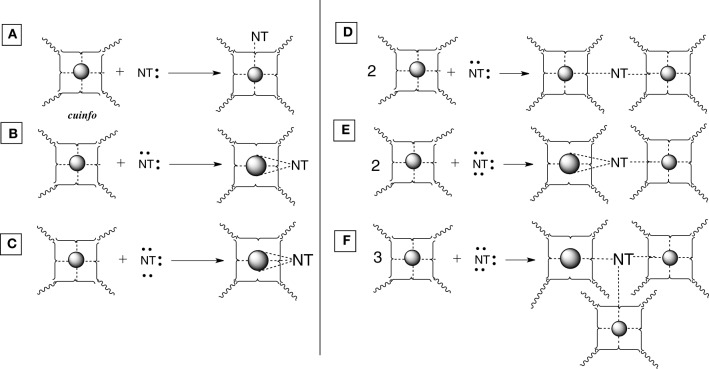
**Various types of [*cuinfo*:NT] complexes. (A)** A monodentate NT replaces one neutrix metal bond. **(B)** A bidentate NT couples (entangles) 2 *cuinfo*. **(C)** A tridentate NT replaces three neutrix metal bonds. **(D)** A bidentate NT entangles two *cuinfo*. **(E)** A tridentate NT entangles two *cuinfo*, via double and single complexing bonds. **(F)** A tridentate NT entangles three *cuinfo*.

We offer an iconography to visualize the formation of *cuinfo* (Figure 3) and their transformation by tagging with NT (Figure 4). To stay within the IUPAC guidelines for chemical notations, the graphic notation previously employed has been slightly modified (Boole, 1853; Marx and Gilon, 2012, 2013). The complexing moieties (ligands) in the nECM are presented as two dots (non-bonding pair of electrons). The metal bonded to the complexing groups in the nECM is indicated by a dotted line (e.g., Figure 3). We have defined an arbitrary unit of cognitive information as *cuinfo.* We call the nECM array with metal complexes: neutrix.

## Examples of metal chelating neurotransmitters (NTs)

The NT can be considered to embody “emotive” signals, in that they elicit emotive physiologic reactions (attention, anxiety, anger, fear, love, pain, etc) that are remembered. Many NT are also effective metal chelators. Chemically, the presence of an NT can “flavor” a *cuinfo*. The resultant ternary complex is more stable, crowned with an emotive tag. Such chelate complexes are reversible depending on their binding strength to a particular *cuinfo* (pK_D_). Some redox and crosslinking reaction can stabilize these.

Mono-, bi- and tridentate complexes of electron-rich NTs with metal-centered *cuinfo* can be conceived (Figure 4). In such mixed complexes, the NT replaces one or more neutrix metal bond also indicated by a dotted line

Of course, cross-linking (from either redox or enzymatic) reactions would render the entire ensemble much more stable, relevant to long term memory.

### Types of NTs

A: Catecholamines: epinephrine (EPI), norepinephrine (NE), dopamine (DA) (Colburn and Maas, 1965; Boggess and Martin, 1975; Chandra et al., 1980; Kroval et al., 2006; García et al., 2012).

Catecholamines are “emotive” neurotransmitters associated with fear, fright, anxiety, all emotions strongly recalled in memory. Physiologically, they elicit responses such an altered heart rate, blood flow, pupil dilation, muscle contraction, etc. Chemically, they comprise an ortho-dihydroxy benzene structure, a potent metal chelating moiety; stored within neuron's vesicles and released upon signaling. The NT of this class, the dopamine (DA), norepinephrine (NE) and epinephrine (EPI) can form ternary complexes with *cuinfo*, generaly described in Figure 6.

The catecholamines present 2 independent chelate centers (the ortho hydroxy benzene and the distal amino terminus) which can bridge two adjacent *cuinfo*, effectively entangling a pair of *cuinfo*, rendering them more stable as well as more identifiable (tagging) for linked (entangled) recall. They permit of emotive memory associated with physiologic reactions.

B: Amino acids (Hughes and Zubek, 1956; White and Rumbold, 1988; Flood et al., 1990; Velez-Pardo et al., 1995; Buhot et al., 2000; Álvarez and Ruarte, 2004; Siegel et al., 2005; Paoletti et al., 2009; Dere et al., 2010; Lesburguères et al., 2011) and other small molecules.

In the same manner, other NT such as glutamine, histidine, and seratonin, which affect numerous physiologic responses (water balance, immune reactions, blood clotting, fever, sweating), as well as emotion, can form mixed complexes with *cuinfo*. A lineup of some NT's capable of adorning the *cuinfo* by chelate complexation is iconographically presented in Figure 7.

C: Metal chelating neuropeptides (Jolles, 1983; Ludlam et al., 1980; Coffman and Dunn, 1988; Marazziti et al., 2006; Neumann, 2007; Wyttenbach et al., 2008; van der Burgt et al., 2009; Guastella et al., 2010; Beets et al., 2012; Garrison et al., 2012; Pitt et al., 2013).

Neuropeptides are an important class of molecular communicators in the central and peripheral nervous systems, acting as neurotransmitters, neuromodulators, and hormones. They also connect the nervous system to other physiological networks regulating breathing, pulse, etc. Many neuropeptides are abundantly expressed in brain regions involved in emotional processing and anxiety behaviors.

Neurotransmitters (NTs) and neuropeptides (NP), having various physiological effects have also been implicated in cognitive functions such as learning and memory. The peptides include corticotropin releasing factor, urocortin, neuropeptide Y, vasoactive intestinal polypeptide, neurotensin, galanin, opioid peptides, tachykinins, nociceptin, oxytocin, vasopressin, and angiotensin. In addition to their many physiological functions, NTs elicit psychic effects on mood (anxiety and depression) and memory.

For example oxytocin is cyclic nona-peptides (9 aa), capable of eliciting numerous physiological responses [lactation, blood coagulation (factor VIII)]. It also affects cognitive functions related to memory as well as to emotions love, mood, appetite, sexual behavior, social behavior. The 3-D structure of complexes of oxytocin with Cu^+2^ and Zn^+2^ and insulin have been described. For example, the groups in oxytocin, which participates in the formation of these complexes can also permit the formation of mixed metal complexes with *cuinfo*. The metal complexing moieties (indicated be arrows in Figure 7) include: the amino terminal Cys^1^ group, the disulfide bond between Cys^1^ and Cys^6^, the phenol group of Tyr^2^, the carboxamide groups of Gln^4^ and Asn^5^, and the terminal carboxamide group of Gly^9^. In addition the peptide bonds constitute multiple metal bonding ligands.

A set of *cuinfo* might be represented as adjacent units adorned by redox or NT tags.

## Discussion

Memory is a mental function that permits recall of past events, to guide future behavior. One could say: “No cognition without memory.” How are different memories assigned value or significance? What are the molecular-scale details? What are the molecular encoders of emotions or feeling (James, 1884; Langer, 1967)?

We point out that the NTs elicit not only physiologic effects but concommitantly elicit psychic effects described as emotions (see Table 2). For the purposes of discussing memory, the NTs can be considered to be the encoders of emotions. With the exception of acetylcholine and muscarine, which both express a tetra-substituted ammonium moiety and are true cations regardless of the pH, the other NTs are all electron donors, capable of forming ternary, metal-centered complexes, described as *cuinfo:NT.*

Consider the computer using binary code. Each bit is anonymous, (100111001110), exhibiting no flavor, color, value or priority, one over the other. The Turing machine computes (performs a series of discrete procedures) inexorably according to the laws of logic, mathematics and communication theory, with no emotional context (Boole, 1853; Turing, 1950) or survival import. The NTs provide the neural system with a novel encoding modality that is missing in binary codes, the emotive option for encoding cog-info, critical for providing value and significance to the memory consolidated from tagged *cuinfo:NT*, aiding survival.

The brain is first and foremost an emotive organ, mentating emotionally with combinatorially large sets of chemical “encoders” (Lehn, 2002, 2012) to ensure survival. Emotions such as fear, anger, love, etc., drive behavior, are the “coins of significance,” which provide a priority value to cog-info, are strongly remembered.

**Figure 5:**
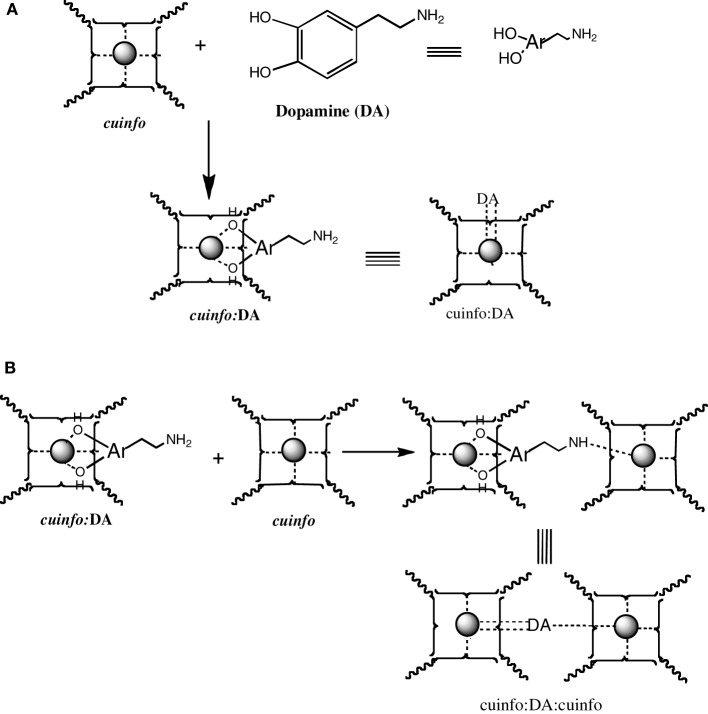
**Formation of mixed dopamine:*cuinfo* complexes. (A)** Formation of ternary *cuinfo* complex with one catecholamine. **(B)** Formation of a entangled dopamine (DA) complex (cuinfo_2_:DA).

**Figure 6:**
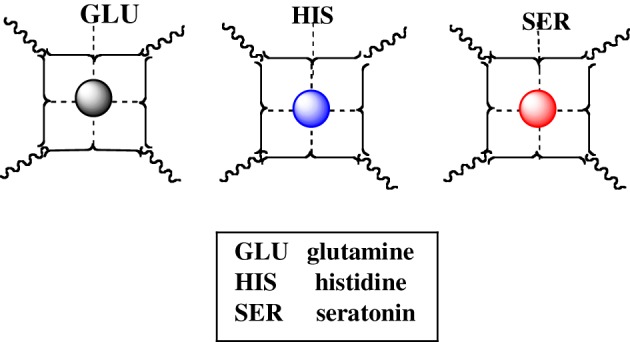
**Icons of *cuinfo* tagged with various neurotransmitters, as ternary complexes**.

**Figure 7:**
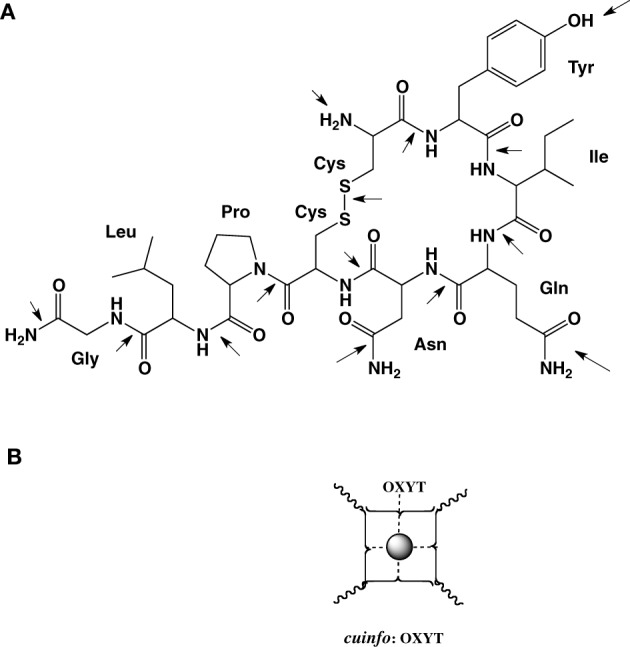
**(A)** Molecular structure of oxytocin (OXYT), with 14 potential metal-complexing moieties marked with arrows. **(B)** Iconographic representation of a monodendate *cuinfo*:OXYT complex.

**Figure 8:**
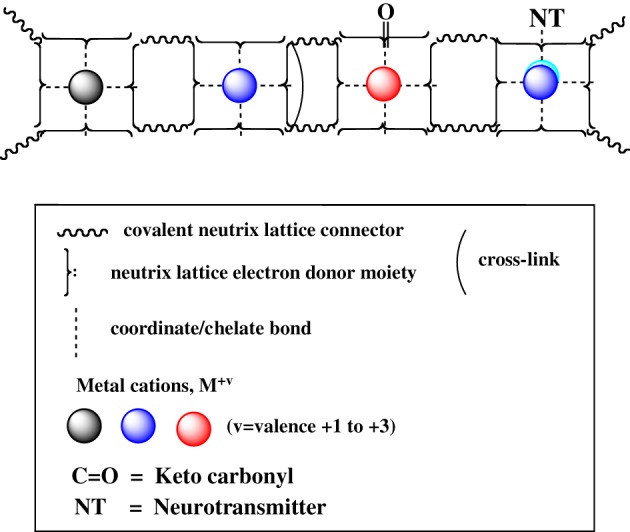
**A set of *cuinfo*, tagged with keto group and a generic neurotransmitter (NT)**.

We may not be able to penetrate the realm of subjective experience, but we can describe the molecular correlates and chemical dispositions of psychical processes (mentation) of which memory is an example. The molecular correlates of emotions could be considered to be encoded by NTs (Tables 1, 2), relatively small molecules that are secreted into the nECM by activated neurons, as part of non-synaptic “chemical signaling” (volume transmission) (Wu et al., 2004; Delgado et al., 2006; Ortega et al., 2007; Syková and Nicholson, 2008; Adlard et al., 2010; Vizi et al., 2010; Kaler, 2011; Sadiq et al., 2012; Trueta and De-Miguel, 2012; Goyal and Chaudhury, 2013; Vizi, 2013). Vesicles containing psychoactive neurometals (Al, Ca, Co, Cu, Fe, Mg, Mn, Zn) are also released by the neuron into the nECM upon firing, combinatorially encoding cog-info as *cuinfo*, ternary (mixed) complexes capable of combing with NTs, described by the iconographic notations in Figures 4–8.

Emotion without memory to guide behavior would be very short-lived… the organism would not long survive. Emotions provide value/priority to (incoming) sensorial cog-info. Emotion and memory are functionally linked phenomena… providing motivational significance (value) to guide adaptive behavior. Emotions could be considered as responses that “flavor” cog-info with value, to aid recall and guide behavior.

All animals need to respond emotionally to a specific circumstance, and to remember the specific situation within the limitations of its evolved capabilities to recall. The NTs are capable of eliciting both physiologic and psychic responses to a significant experience. Thus, they can affect behavior and also imprint *cuinfo* with emotive tags, to enable “prioritized recall,” enabling survival. The above-described *tripartite* mechanism with NTs, brings emotion-laden mental sensibility into the compass of biochemical fact, applicable to all neural creatures exhibiting memory.

### Conflict of interest statement

The authors declare that the research was conducted in the absence of any commercial or financial relationships that could be construed as a potential conflict of interest.
